# Assessing the True Impact of Recurrent Fractures on Fracture Risk

**DOI:** 10.1359/jbmr.090814

**Published:** 2009-08-17

**Authors:** Bart L Clarke, Sundeep Khosla

**Affiliations:** 1 Division of Endocrinology, Diabetes, Metabolism, and Nutrition, Mayo Clinic and Mayo Foundation Rochester, Minnesota, USA; 2 Endocrine Research Unit, Mayo Clinic and Mayo Foundation Rochester, Minnesota, USA

Fractures continue to be an important public health problem. A total of ∼9 million osteoporotic fractures were reported worldwide in 2000,(1) with ∼20% of these being hip fractures. The importance of these fractures is shown by their association with increased mortality,(2) decreased quality of life,(3) and substantial direct and indirect costs to the health care systems of many countries.(4)

The major strategy to minimize the burden of osteoporotic fractures on society has been to identify and treat high-risk individuals before they fracture. The challenge has been to accurately assess fracture risk prospectively. The most common way fracture risk has been assessed clinically is by using DXA BMD. The WHO currently defines BMD results as normal when T-scores are greater than −1.0, osteopenic when T-scores are between −1.0 and −2.5, and osteoporotic when T-scores are less than −2.5. Normal BMD is interpreted as indicating low risk of fracture, osteopenia as indicating medium risk, and osteoporosis as indicating high risk. This classification scheme clearly identifies those individuals with the highest risk of fracture, but because the greatest number of fractures occur in individuals with osteopenia or normal BMD, because of the much larger numbers of these individuals in the population, it is unable to prospectively identify many individuals who subsequently fracture.(5–8) As a result, a number of studies have attempted to identify risk factors in addition to BMD that would improve the ability to predict fractures in those with normal or osteopenic BMD.

Multiple risk factors have been identified for osteoporotic fracture. One of the strongest and most consistent risk factors across multiple studies in different populations is personal history of low-trauma fracture.(7,9,10) However, most cohort studies have excluded repeat fractures in the same individual from consideration during the study interval in the effort to estimate incident fractures. Excluding repeat fractures in the same individual reduces population fracture estimates below the true prevalence in the population. This is an important consideration because incident fractures increase subsequent fractures over at least the next year.(11,12) Accurate estimates of population fracture prevalence should logically include repeat clinical fractures in the same individuals who experience them.

It is in this context that the paper by Langsetmo et al.(13) in this issue of the *JBMR* is of particular interest. This study sought to quantify how many low-trauma fractures were first fractures and how many were repeat fractures in subjects randomly selected from the Canadian population for the Canadian Multicentre Osteoporosis Study (CaMOS). The CaMOS study includes 2179 men and 5269 women 50–90 yr of age at recruitment. The study cohort used for this study excluded 51 men and 83 women because they did not have follow-up data and 245 men and 698 women because they did not have baseline BMD. All low-trauma clinical fractures that occurred in this cohort over 8 yr of follow-up were included in this study and classified as either first or repeat fractures. Analyses were stratified by sex, age, BMD risk categories, and vertebral deformity status. BMD risk categories were defined as normal, osteopenia, or osteoporosis, based on current WHO T-score definitions.

The study cohort at risk for first fracture included 2044 men with a mean follow-up of 5.8 yr and 4612 women with a mean follow-up of 6.0 yr. There were 95 fractures in the men and 344 fractures in the women. The cohort at risk for repeat fracture had 230 men with a mean follow-up of 4.8 yr and 1001 women with a mean follow-up of 5.1 yr. There were 33 fractures in the men and 233 fractures in the women. The study therefore reported a total of 128 incident fractures in men and 577 fractures in women over the 8 yr of follow-up.

Of these fractures, 25% were repeat fractures in the men and 40% repeat fractures in the women. Just more than one half of the first fractures occurred in subjects with osteopenia (58% in men, 54% in women) and just less than one half of the repeat fractures occurred in subjects with osteopenia (42% in men, 47% in women). Most first fractures occurred in subjects with osteopenia, whereas repeat fractures were evenly divided between the normal, osteopenic, and osteoporotic categories in men and between the osteopenic and osteoporotic categories in women. Subjects with osteoporosis contributed 21% of the total fracture burden in men and 39% of the fracture burden in women.

The estimated age-adjusted prevalence of fracture risk determined by this study indicated that an estimated 4.6% of men and 9.3% of women would have a history of low trauma fracture after age 40, if a population had the same sex-specific age distribution as the 2001 Canadian census population between 50 and 90. An additional 3.8% of men and 9.6% of women in this population would have had an osteoporotic BMD but no history of low-trauma fracture.

The study also showed that both first and repeat fractures increased by 60–70% per decade of age in both men and women and that the incidence of repeat fracture was significantly higher than incidence of first fracture within each 10-yr age group. The incidence of repeat fracture ranged from 2- to 4-fold higher than the incidence of first fracture within a given BMD category for both men and women.

The study showed that vertebral deformity and previous low-trauma fracture were independent predictors of fracture. In both men and women with grade 2 or higher vertebral deformity, the incidence of first fracture was increased, but in those without vertebral deformity, the risk was decreased. This was also true for repeat fractures.

The authors concluded that repeat fractures contribute significantly to the overall fracture burden and that the incidence of repeat fractures is independent of BMD. This study showed that subjects with a combination of prior low-trauma fracture and another risk factor were at especially high risk of future fracture.

This study is important because it highlights the danger that true population fracture prevalence will be significantly underestimated if repeat clinically significant low-trauma fractures are eliminated from consideration. In the CaMOS population, one quarter of the overall fractures in men and 40% of overall fractures in women were repeat fractures. This observation emphasizes the importance of previous personal history of fracture in predicting future fracture risk, independent of BMD. The study supports the contention that, whereas BMD in the osteoporotic range is a good predictor of fracture in patients who have not yet had low-trauma fractures, a prior low-trauma fracture is a sign of skeletal fragility regardless of BMD.

The finding that the majority of overall fractures in the CaMOS study population occurred in men and women with osteopenia is consistent with other studies of postmenopausal women.(5–8) This study further found that the majority of first clinical low-trauma fractures occurred in those with osteopenia, whereas repeat clinical low-trauma fractures occurred with equal frequency in women with osteopenia or osteoporosis and in men with osteopenia, osteoporosis, or normal BMD. The significance of these findings is that individuals with osteopenia have increased risk of both first and repeat low-trauma fractures. Individuals with osteopenia who have had a first clinical low-trauma fracture should be viewed as being at high risk of subsequent fracture, regardless of their relatively reassuring BMD results.

The differences in results seen between men and women with osteopenia in this study are notable. Men and women with osteoporosis had similar rates of first and repeat fractures. Most fractures in women occurred in those with osteoporosis or prior fracture, but the majority of fractures in men occurred in those with osteopenia or normal BMD and without previous fracture. These findings suggest that BMD results in men have different implications than in women, that risk factors for fracture in men may be different than those in women, and that cost-effective interventions for men based on BMD may be different from those in women.

This study confirmed that the risk of repeat clinical low-trauma fracture is about twice the risk of first clinical low-trauma fracture after stratification by age and BMD, similar to what other studies have reported.(7,12,14) Vertebral deformity was also found to be a risk factor for fracture, with repeat clinical fracture at least twice the incidence of first clinical fracture after stratification for vertebral deformity.

In summary, the findings of the paper by Langsetmo et al.(13) provide further evidence of the significance of previous personal history of low-trauma fracture in determining subsequent fracture risk. Previous personal history of low-trauma fracture increases risk of subsequent fracture, particularly in patients with osteopenia, independent of BMD results. For the practicing physician, this study highlights the importance of obtaining a careful history regarding previous fractures, perhaps complemented by vertebral fracture assessment by DXA(15) or spine X-rays (when indicated) to define preexisting fractures.
